# Spatiotemporal network motif reveals the biological traits of developmental gene regulatory networks in *Drosophila melanogaster*

**DOI:** 10.1186/1752-0509-6-31

**Published:** 2012-05-01

**Authors:** Man-Sun Kim, Jeong-Rae Kim, Dongsan Kim, Arthur D Lander, Kwang-Hyun Cho

**Affiliations:** 1 Department of Bio and Brain Engineering, Korea Advanced Institute of Science and Technology (KAIST), Daejeon, 305-701, Republic of Korea; 2 Department of Mathematics, University of Seoul, Seoul, 130-743, Republic of Korea; 3 Department of Developmental and Cell Biology and Department of Biomedical Engineering, Center for Complex Biological Systems, University of California, Irvine, Irvine, CA, 92697-2300, USA

## Abstract

**Background:**

Network motifs provided a “conceptual tool” for understanding the functional principles of biological networks, but such motifs have primarily been used to consider static network structures. Static networks, however, cannot be used to reveal time- and region-specific traits of biological systems. To overcome this limitation, we proposed the concept of a “spatiotemporal network motif,” a spatiotemporal sequence of network motifs of sub-networks which are active only at specific time points and body parts.

**Results:**

On the basis of this concept, we analyzed the developmental gene regulatory network of the *Drosophila melanogaster* embryo. We identified spatiotemporal network motifs and investigated their distribution pattern in time and space. As a result, we found how key developmental processes are temporally and spatially regulated by the gene network. In particular, we found that nested feedback loops appeared frequently throughout the entire developmental process. From mathematical simulations, we found that mutual inhibition in the nested feedback loops contributes to the formation of spatial expression patterns.

**Conclusions:**

Taken together, the proposed concept and the simulations can be used to unravel the design principle of developmental gene regulatory networks.

## Background

To uncover the governing principles underlying complex biological processes, it is important to understand the relationship between topological structures and the dynamical characteristics of gene regulatory networks [1-4]. One promising method of investigation is to disassemble the large regulatory network into its more basic, constituent building blocks called network motifs, which recur within a network much more often than expected in random networks. Network motifs are considered to have been evolutionarily selected because of their functional advantages [5].

Most previous studies have identified network motifs of biological networks by implicitly assuming that all the links in a network can be active or working at the same time. However, such approaches may not be applicable to developmental networks where all genes and interactions do not operate simultaneously due to spatial and temporal variations. Some network motif approaches have partially considered spatial or temporal information on biological networks [6,7]. Papatsenko analyzed the dynamics of network motifs for a spatial stripe pattern formation, only in early embryogenesis [7], while Kim et al. explored the dynamics for temporal network motifs [6]. Nevertheless, patterns of spatiotemporal variations in gene regulatory networks have not yet been explored.

In this paper, we propose a novel concept called the “spatiotemporal network motif,” which is a sequence of network motifs in sub-networks that are spatiotemporally active. These network motifs are constructed by re-organizing the regulations between spatiotemporally expressed genes.

We applied this approach to the developmental gene regulatory network of *D. melanogaster*. First, we identified a spatio-temporal sequence of network motifs which change according to developmental stages and regions. Then, we analyzed the pattern of spatio-temporal network motifs and their dynamics (we only considered three-node network motifs for simplicity and for a concise illustration of the method). As a result, we found that the most frequently observed structure in the spatiotemporal network motif pattern is the feed-forward loop structure. This result implies that signal-processing via feed-forward loops is required throughout all of the development stages [6,8]. Another important network motif that we identified was nested feedback loops, where one feedback loop is nested inside another feedback or feed-forward loop. Such nested feedback loops were considered necessary for the development of a central nervous system, as they should be stable and robust against both noise and small perturbations [9]. This result suggests that nested feedback loops might play an important role in the elaborate regulation of developmental processes. Interestingly, we found that most nested feedback loops had mutual inhibitory structures. Through mathematical simulations, we showed that such mutual inhibitory structures can enable exclusive spatial expression of gap genes.

Taken together, the proposed concept and the simulations can reveal time- and region-specific biological traits in dynamic processes such as the developmental gene regulatory network, and can be widely used to investigate the relationship between dynamic network structures and their regulatory functions.

## Results

### Identification of spatio-temporal network motifs

**Figure 1:**
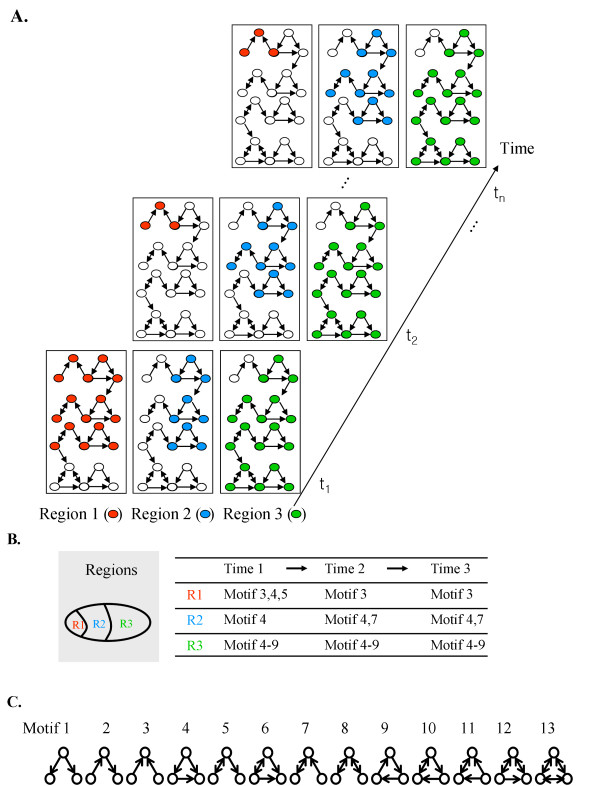
**Conceptual illustration of a spatiotemporal sequence of network motifs.** (**a**) A network featuring spatiotemporal variations. Red, blue, and green circles denote the nodes in active sub-networks of regions 1, 2, and 3, respectively. Filled and unfilled circles denote active and inactive nodes, respectively. (**b**) Illustration of a spatiotemporal sequence of network motifs in nine active sub-networks. Each region-specific network has its own network motifs and the occurrence of the network motifs changes over time. (**c**) Thirteen possible three-node sub-network structures.

Gene expression is controlled by spatiotemporally active sub-networks of a gene regulatory network (Figure 1a). In order to study the biological traits of dynamic systems such as developmental processes, we need to analyze the structural characteristics of such spatiotemporally active sub-networks. To this end, we propose a new concept called “spatiotemporal network motifs,” which can be used to reveal region- and time-specific biological traits at a system level and to study the relationship between topological structures and the functional roles of spatiotemporally active sub-networks.

Let us consider an example network with gene expression data measurements taken in three regions (R1, R2 and R3) at three time points (T1, T2 and T3), as shown in Figure 1b. In this case, we should consider nine active sub-networks. Figure 1b shows the network motif pattern of the nine active sub-networks. The network motifs of the R3 sub-network do not vary as time evolves, whereas the patterns of network motifs in the R1 or R2 sub-networks vary. In addition, at each fixed point in time, the network motifs of the active sub-networks were different in all three regions. For instance, Motif 3 was found in R1, but not in other regions. This suggested that Motif 3 is necessary for some particular biological function only found in R1. Likewise, we can use the proposed approach to identify network structures that are necessary at some specific region and time.

### Unravelling region- and time-specific biological traits of developmental processes

**Figure 2:**
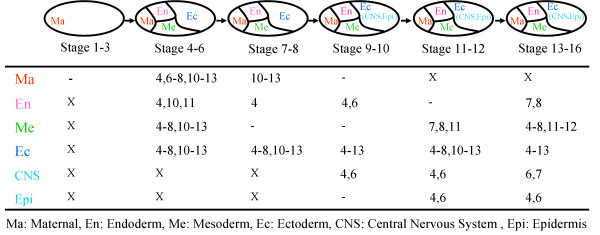
**A spatiotemporal sequence of network motifs of the*D. melanogastor*developmental gene network.** ‘X’ denotes the region where we could not apply our network motif identification due to the absence of active sub-network and ‘-’ denotes the region where we did not identify any network motif. ‘(CNS, Epi)’ in the ellipse indicates that the two regions CNS and Epi are differentiated from Ec and located inside Ec.

We applied the proposed approach to the developmental gene regulatory network of *D. melanogaster*, since developmental processes should be driven by tightly coordinated spatio-temporal patterns of gene expression. For this case study, we collected gene expression data measured at six developmental stages from the BDGP (Berkeley Drosophila Genome Project) database [10] and considered six body parts: Maternal (Ma), Endoderm (En), Mesoderm (Me), Ectoderm (Ec), Central Nervous System (CNS), and Epidermis (Epi). There were 30 active sub-networks in all (Figure 2), which we used to identify network motifs. We were able to identify network motifs from 19 active sub-networks, meaning that the remaining 11 active sub-networks do not contain any network structures that occur more often than in random networks. These results cannot be obtained from conventional network motif analysis. For instance, Motifs 3, 4, 6, 11, 12, and 13 were identified in the static network [6], which is simply the union of all the possible regulations between genes. However, not every component in the entire network is active throughout the overall developmental stages and regions. With our approach, we can find new network motifs that cannot be identified from conventional motif analysis. The temporal network motif approach [6] is more advanced, in that it expands to include temporal changes. Nested feedback loops were identified at late stages by reconstructing time varying networks, but our approach is the first to successfully identify such loops as important network motifs in overall developmental stages. Nested feedback loops were required for pattern formation at early embryogenesis, and for neurogenesis at middle embryogenesis. Taken together, the proposed concept is more practical than previous approaches for analyzing developmental gene regulatory networks.

In Table 1, we summarized body parts, the corresponding network motifs, the triple genes comprising the network motifs, and the Gene Ontology (GO) terms of three periods (early, middle, and late embryogenesis). We investigated whether the genes in the spatio-temporal network motifs possessed the GO terms related to each developmental stage. In the early period of embryogenesis (Stages 1–6), a triplet of genes—*bcd, hb,* and *kni*—were related to the zygotic determination of anterior/posterior axis (GO: 0007354). The *bcd* gene encodes the morphogen responsible for the head structure and the gradient of *hb* and *kni* is particularly important in establishing the axis of the body. Another triplet of genes—*eve*, *en*, and *zen*—were expressed in the dors ectoderm tissue and in the anlage in statu nascendi (*AISN*) anatomical structure. The gene *en* is one of the segment polarity genes and is expressed in cells where *eve* is highly expressed, while *zen,* a dorsalizing gene, is inhibited by *eve* and *en* in the ventral region.

**Table 1:** We have summarized body part(s), network motifs, triple genes, and GO terms for three periods (early, middle, and late stages)

**Periods**	**Body parts**	**Network motif**	**Triple** **genes**	**GO term (*p*-value)**
Early	Maternal	Motif 11	*bcd, hb, kni*	GO:0007354 (3.26e-06)
	dors ectoderm AISN	Motif 12	*Kr, hb, gt*	GO:0007354 (3.26e-06)
	vent ectoderm AISN			
	procephalic ectoderm AISN			
	dors ectoderm AISN	Motif 4	*eve, en, zen*	GO:0016564 (8.55e-05)
Middle	Post endoderm PR	Motif 4	*tll, Kr, salm*	GO:0045165 (2.10e-03)
	hindgut A	Motif 4	*tll, hb, salm*	GO:0001708 (5.11e-05)
	vent nerve cord PR P3	Motif 12	*cas, Kr, nub*	GO:0007402 (4.31e-08)
	vent nerve cord PR P3	Motif 12	*Kr, hb, nub*	GO:0007402 (5.29e-08)
	vent ectoderm PR	Motif 4	*hb, en, eve*	GO:0001709 (2.79e-04)
Late	clypeo-labral PR	Motif 6	*odd, prd, en*	GO:0007365 (9.40e-06)
	vent epidermis PR			
	vent epidermis PR	Motif 6	*nkd, en, wg*	GO:0014016 (8.88e-06)
	clypeo-labral PR	Motif 6	*slp1, en, wg*	GO:0035289 (6.14e-07)
	vent epidermis PR			
	dors epidermis PR			
	brain	Motif 12	*Kr, hb, nub*	GO:0007402 (5.29e-08)
	vent nerve cord			
	brain	Motif 7	*gt, Kr, hkb*	GO:0007354 (3.26e-06)
	brain		*hb, Kr, Nos*	GO:0001709 (3.21e-04)

**Abbreviation**: *bcd (bicoid), cad (caudal), cas (castor), en (engrailed), eve (even slipped), gt (giant), hb (hunchback), kni (knirps), Kr (Kruppel), nkd (naked cuticle), Nos (nanos), nub (nubbin), odd (odd skipped), prd (paired), salm (spalt major), slp (sloppy paired), tll (tailless), wg (wingless), zen (zerknullt)*

**GO terms**: GO:0007354 zygotic determination of anterior/posterior axis, embryo, GO:0016564 transcription repressor activity, GO:0045165 cell fate commitment, GO:0001708 cell fate specification, GO:0007402 ganglion mother cell fate determination, GO:0007365 periodic partitioning, GO:0014016 neuroblast differentiation, GO:0035289 posterior head segmentation.

In the middle period of embryogenesis (Stages 7–10), one of the key events is the completion of gastrulation. During gastrulation, the morphology of an embryo is rearranged to form the three germ layers: ectoderm, mesoderm, and endoderm [11]. Another triplet of genes—*tllKr,* and *salm*—were expressed in the endoderm tissue. The gene *tll* is one of the gastrulation genes and *salm* is well-known to influence the development of gut. Another triplet of genes—*cas hbKr*, and *nub*—were related to determining the fate of the ganglion mother cell (GO: 0007402), and were expressed in the ventral nerve cord tissue. This suggested that the identified genes might influence the development of the central nervous system (CNS). The midline precursors undergo a synchronous cell division to give rise to 16 midline progenitor cells per segment. Then, until stage 12, these 16 midline cells go through cell shape changes, cell division, and differentiation to form the midline primordium (*PR*) [12].

In the late period of embryogenesis (Stages 11–16), mature CNS midline cells were completed by stage 13, resulting in the development of the brain organ. Genes related to epidermis (*odd, prd, en, slp1*, and *wg*) were identified in vent and dors epidermis tissues. Epidermis is the outer layer of skin, which originates from the ectodermal cells covering the embryo. In Table 2, we summarized the result of GO analysis on various tissues, which shows that the genes involved in these network motifs are related to the late period of embryogenesis.

**Table 2:** Illustration of the GO analysis for late embryogenesis

**Region**	**GO Term**	***p*-value**
Ectoderm	GO:0048513 organ development	1.81e-34
	GO:0007423 sensory organ development	1.81e-11
Mesoderm	GO:0007507 heart development	1.22e-13
	GO:0061061 muscle structure development	6.00e-09
	GO:0007498 mesoderm development	1.47e-08
CNS	GO:0014016 neuroblast differentiation	2.28e-15
	GO:0007400 neuroblast fate determination	8.48e-14
	GO:0007419 ventral cord development	3.09e-10
Epidermis	GO:0008544 epidermis development	4.83e-03

### Dynamical and structural analysis of the gap gene network

We found that nested feedback loops (Motif 6, 10, 11, 12 and 13) were frequently observed in the developmental network, especially in the gap gene network. The combination of feed-forward loops and feedback loops can induce an emergent property [6]. For example, in stages 4–16, Motifs 4–8 and 10–13 are commonly observed in the ectoderm. These stages include the sequential development of the central nervous system and epidermis in the ectoderm tissue. These processes are both important in ectoderm-specific development, and are considered to require stable and robust regulation against both noises and small perturbations [9]. The network of *Drosophila* neurogenesis also features nested feedback loops [13]. Nakajima et al. suggested that nested feedback loops composed of *hbKrnub*, and *cas* are precisely regulated by three different kinds of links. The authors also found a minimum network which can reproduce the sequential expression pattern of the four genes. However, the minimum network is less robust against parameter variations than the original network with nested feedback loops. Hence, we conclude that the nested feedback loops induce an emergent property for the elaborate and robust regulations of developmental processes.

**Figure 3:**
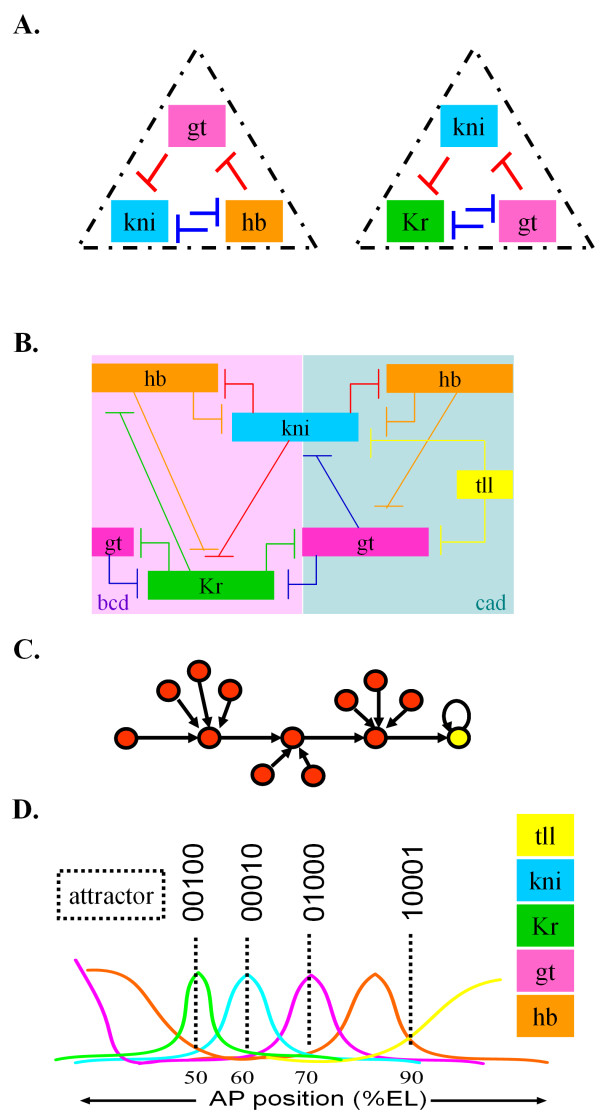
**The structure and dynamics of the gap gene network.** (**a**) Nested feedback loops with two different kinds of regulations. Different kinds of regulations generate precise spatial expressions and permit shared spatial expressions. (**b**) Gene regulatory network of gap genes. This figure shows that the gap genes are activated or repressed by maternal genes, and are expressed in two broad domains (*bcd* and *cad* domain) along the AP axis (Jaeger 2009). (**c**) Conceptual illustration of an attractor. Dynamic trajectories of the gene states (red nodes) flow to the fixed point attractor (yellow node). (**d**) Spatial distribution of the five participating gap genes along the anterior-posterior (AP) axis (where 0% EL is the anterior pole), with gap genes plotted separately. The attractors are represented by dotted lines in 50, 60, 70 and 90% EL, respectively.

Interestingly, we observed that most of the nested feedback loops contained mutual inhibition structures. We found that gap genes were frequently observed in the nested feedback loops of stages 4–6, and most links of the gap gene network are mutual inhibitions (Figure 3a). In addition, for three nested feedback loops (Motifs 10, 11, and 12), we calculated the percentage of gap gens out of the genes that constitute each network motif with mutual inhibition, and found a significant proportion of gap genes (52% for Motif 10, 43% for Motif 11, and 56% for Motif 12). The mutual inhibition structure plays an important role. For example, positive feedback loops consisting of mutual inhibition structures are associated with developmental switches or the implementation of positional information [14-17]. In particular, the mutual inhibition structure in developmental gene regulatory networks is associated with inducing exclusive spatial expression of gap genes [18]. It is well known that this mutual inhibition results in the precise placement of stripes, and also permits overlaps of expression between adjacent gap genes [19,20].

Why are the gap genes connected with each other through such a complex structure? In order to understand the dynamics of the complex network structure, we performed Boolean simulations based on the gap gene network (Figure 3b). Then, we identified attractors, where an attractor means a set towards which a dynamical system evolves over time (Figure 3c). Each attractor can be mapped into the lineage-associated transcription factor activities [21-23] and can represent a developmental cell fate. For example, in the model of myeloid progenitor cell differentiation, there are two attractors where GATA-1 and PU.1 are exclusively expressed, which correspond to erythroid/megakaryocyte and myeloid-monocytic fates, respectively [21]. Here, an attractor is expressed as a combination of the digits 1 and 0, where 1 denotes an expressed state of a gene and 0 denotes an unexpressed state of a gene in a specific region. As a result of the Boolean simulation for five gap genes (*hb, gt, Kr, kni,* and *tll*), we identified eight attractors within the gap gene network (Additional file 1: Figure S3 and Table S1). The gap genes are activated or repressed by the maternal effect genes (*bcd* and *cad*) (Figure 3B). The concentration of the protein *bcd* is high in the anterior region [24], while the concentration of the protein *cad* is high in the posterior region. The spatial-specific concentration of maternal effect genes affects the spatial-specific concentration of gap genes, which in turn results in distinct steady states which correspond to attractors. The attractors can be classified into two classes: attractors corresponding to the case where only one out of five gap genes is expressed (attractors 01000, 00100, 00010, 00001 and 10000) and attractors corresponding to the case where more than one gene are expressed (attractors 10001 and 00101). For example, an attractor 00100 means that only *Kr* is expressed and the rest of genes are unexpressed. It is well known that among gap genes, only *Kr* is expressed in the central region [25-27] . Hence, the attractor 00100 corresponds to a state of gap gene expression at a special region (i.e., the body part of 50% egg length (EL). Likewise, another attractor 10001 corresponds to the spatial expression pattern in the posterior pole region (Figure 3d) where only two genes, *hb* and *tll*, are expressed [28,29], and the rest of genes are unexpressed. Similarily, two other attractors 00010 and 01000 also correspond to a set of spatial expression patterns of gap genes. Hence, we conclude that the complex structure of the gap gene network with nested feedback loops contributes to the formation of spatial expression patterns.

**Figure 4:**
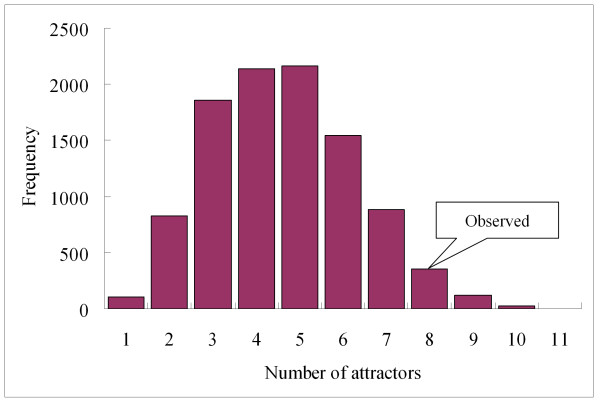
**Distribution of the number of attractors of the gap gene network.** The frequency of attractors (“Observed”) compared to that of 10,000 random networks with the same topological structure as the gap gene network, except for the signs of the links.

The gap gene network has eight attractors. Do the mutual inhibition structures in the network help to induce many attractors? To answer this question, we counted the number of attractors for 10,000 random Boolean networks whose topological structures are the same as the gap gene network, except for the signs of the links. Figure 4 shows the distribution of attractors. Only 1.4% of the random networks have more than eight attractors. In other words, the network with mutual inhibitions has a significantly large number of attractors. We further investigated the number of attractors while reducing the number of mutual inhibitions of the network. In order to keep the total number of links in the network same while removing mutual inhibitions, we have changed the signs (from inhibition to activation) of the links in the mutual inhibitions. We found that the average numbers of attractors were 6.80, 6.07, and 5.98 when we removed one, two, and three mutual inhibitions, respectively. This indicates that the number of attractors is positively correlated with the number of mutual inhibitions. Therefore, we can say that eight attractors in the network are attributable to mutual inhibition. Hence we conclude that the gap gene network has evolved to induce a large number of attractors which correspond to various developmental states.

## Discussion

The development of multi-cellular organisms relies on the coordinated spatiotemporal regulation of gene expressions. To unravel the organizing principles of developmental gene regulatory networks, it is crucial to understand the relationship between the structure and function of spatiotemporal subnetworks. However, thus far, such spatiotemporal networks have not been investigated. Hence, we proposed a new concept called a “spatiotemporal network motif,” which is a sequence of network motifs in time and space, and we applied this concept to analyze the developmental gene regulatory network of *D. melanogaster.* We found that the results of our approach coincide with spatially specific processes in early, middle, and late embryogenesis.

We also identified patterns of spatiotemporal network motifs and analyzed the relationship between network structures and their biological functions (Figure 2). We found that the most frequently observed structure in the spatiotemporal network motif pattern was the feed-forward loop structure (Motif 4) (79% for 19 sub-networks). This finding is also well supported by recent studies: Motif 4 is found ubiquitously in the *D. melanogaster* gene regulatory network [6]; Motif 4 is the core structure of the *D. melanogaster* gene regulatory network [30]; Motif 4 plays an essential role in the *D. melanogaster* central nervous system [31]. This structure, with various regulation types, has several important functions in a biological network, such as detecting persistent signals, generating pulse, and accelerating response [5]. For example, in the feed-forward loop consisting of the three genes *eve, en*, and *hb,* the genes are related to specifying cell fate (GO: 0001708) and commitment (GO: 0045165). These triple genes form a coherent type-1 of Motif 4, such that *eve* and *en* are activated by *hb*, and *en* is activated by *eve* in ectoderm tissue at stages 4–6. In this developmental process, the dynamics of the coherent type-1 [32] of Motif 4 can be used as a persistent signal detector, which enables the system to respond only to persistent signals while neglecting short-term signals. This means that developmental processes related to cell fate must be robust in relation to noises, and explains how the developmental network deals with noises via the structure of the coherent type-1 of Motif 4.

Interestingly, we found that nested feedback loops were frequently observed in the gap gene network and most of the nested feedback loops contain mutual inhibition structures. Based on Boolean simulations, we showed that the gap gene network has a significantly large number of attractors (eight attractors) and such many attractors in the network are attributable to mutual inhibition. Hence we infer that the gap gene network might have evolved to induce a large number of attractors (by increasing the number of mutual inhibitions) which correspond to various developmental states.

The interlinked incoherent feed-forward loop structure is a key regulatory structure for stripe formation at 4–6 stages in the maternal region [7] and we identified this network motif (ID 6) at the same spatiotemporal developmental stages. The triple genes (*gt, Kr* and *eve*) of the network motif ID 6 are also consistent with the previous study [6]. In addition, it is well-known that the feed-forward loop is a crucial structure to DV (Droso-Ventral) axis formation at 4–6 stages in the maternal region [33] and we also identified this network motif (ID 4) at the same spatiotemporal stages. From these, we conclude we could infer the design principles of *Drosophila* development in a holistic manner using our approach.

Network motifs cannot uniquely determine the whole dynamical properties of a regulatory network. In general, the dynamics of a regulatory network depends on multiple factors such as initial conditions, cellular environments, and randomness [34,35]. However, some particular dynamical properties can be determined by certain network structures [36]. For instance, bistable switching cannot be realized without a positive feedback loop in the regulatory network. So, understanding the relationship between network structures and dynamics may still be useful as we can infer some possible dynamical characteristics of a network from its structure. The proposed approach guides us to find specific network motifs (e.g., positive feedback) at a specific spatiotemporal stage and therefore we can estimate possible dynamical properties (e.g., bistable switch) related to the identified network motifs. Taken together, our approach is useful to infer developmental functions of spatiotemporally varying cells based on identification of network motifs.

Most of the previous studies identified network motifs of the whole regulatory networks integrated from various literatures without considering particular biological contexts (e.g., environmental conditions, developmental stages, etc.). The key difference of our study from the previous ones is the identification of network motifs depending on active sub-network assuming that only part of genes may express under some particular spatiotemporal condition. Such a concept has not been proposed so far. This concept provides us (time- and space-) varying patterns of network motifs in terms of time and region simultaneously. For instance, we can find out many types of network motifs at the 4–8 stages, while there is no network motif at the 9–16 stages in the maternal region (Figure 2). From this, we can infer that the maternal region requires more complex regulation through several types of network motifs at the former stages compared to the latter stages.

The topological structures of the network motifs that we discovered in this study are not new by themselves. However, the sequence of time- and space-varying network motifs is new. Furthermore, we could associate the dynamical properties of identified network motifs and spatiotemporal developmental processes of *Drosophila*. For instance, it is well known that the major developmental process at 4–6 stages is differentiation, but there is no differentiation at 1–3 stages in the maternal region. Interestingly, we found the mutual inhibition network motif (ID 8), a key network motif for the differentiation process, at 4–6 stages but not at 1–3 stages. Together, we can infer a specific developmental process at a specific developmental stage from the dynamical properties of the identified network motifs. Furthermore, the presented approach provides us with a useful and single framework in which we can investigate the whole developmental process in a comprehensive view.

## Conclusions

We proposed a novel concept called the “spatiotemporal network motif,” which is a sequence of network motifs in sub-networks that are spatiotemporally active. Since the proposed approach is based on the network motif framework, many important issues must still be addressed, including the reliability of the constructed network and the justification of DEG selection. Nonetheless, the proposed approach can provide a good framework for improving our understanding of developmental processes and identifying key regulatory processes. Also, by applying the proposed approach to a developmental network, we can gain new insights into the organizing principles of a developmental network whose structures change spatially and temporally, and can be widely used to investigate the relationship between dynamic network structures and their regulatory functions.

## Methods

### Identification of active sub-networks

In order to reconstruct active sub-networks from a whole gene regulatory network, we need two kinds of information: an integrated gene regulatory network and information on the differentially expressed genes (DEGs) in each region and stage. To acquire the DEGs, we incorporated spatiotemporal information from the BDGP database (see the following subsection—“The BDGP database for spatio-temporal information” for details) [10]. The gene regulatory network data of *D. melanogaster* were retrieved from the TRANSFAC database [37], which is a manually curated transcriptional network database. The network consists of 155 nodes and 377 links (see Additional file 2). To identify which sub-network is active at each time point and region, we used the following information: if two genes that share an edge in the gene regulatory network are also included in the DEG set, then those two genes and the connecting link constitute the active sub-network. By repeating this procedure, we obtain a set of spatio-temporally active sub-networks (Additional file 1: Figure S2).

### Microarray data and BDGP database for spatiotemporal information

In this dataset, the expression levels were measured at 22 sequential time points for 16 h (stages 1–16) during embryogenesis [38]. All the data were measured with the time interval of 1 h and the sampling time points during the first 6 h were selected with 1-h overlapping period (from 1.5-2.5 h to 6.5-7.5 h). All the data were verified through the morphological trait of each developmental stage [38]. Therefore, the time points we used were not arbitrarily chosen, but carefully selected to represent molecular profiles of each developmental stage. From the given time-series microarray data, we transformed the time window into the corresponding developmental stage using FlyMove [39].

The BDGP database contains 97,732 digital photographs and expression data for 7,152 genes. The images representing gene expression patterns were classified according to pre-defined stage range annotations. For temporal information, the BDGP data for the first 16 stages of embryogenesis were divided into six stage ranges (stages 1–3, 4–6, 7–8, 9–10, 11–12, and 13–16). For annotation information (http://bigbrain.lbl.gov/cgi-bin/benb/vocab_selector.pl?noreturn=1http://www.fruitfly-.org/ex/Annotation.htm), we obtained a controlled vocabulary to annotate gene expression patterns during embryogenesis. For spatial information, we used the annotations to group developmental structures into six organ systems [40]: Maternal (Ma), Endoderm (En), Mesoderm (Me), Ectoderm (Ec), Central Nervous System (CNS), and Epidermis (Epi). Each body part was also subdivided into four types of anatomical structures (with a suffix): anlage in statu nascendi (AISN); anlage; primordium (PR) (primordium usually develops from an anlage, and can give rise to one or more differentiated organs); differentiated organs. Additional file 1: Figure S2 summarizes the procedure for selecting the DEG sets, and the results are provided in Additional file 2. The procedure for reconstructing spatiotemporally active sub-networks (for details, see [6]) is illustrated in Additional file 1: Figure S3.

### Motif enrichment analysis

In order to identify and visualize network motifs using MAVisto, we generated 1,000 randomized networks by randomly reshuffling links while preserving the in- and out-degree of each node in the original network [41]. Next, we identified two-node and three-node motifs for the gene regulatory network of *D. melanogaster* (using 0.01 as the cut-off threshold for network motifs).

### GO (gene ontology) analysis

GO annotation is used to indicate gene traits. GO analysis is a useful tool for both small- and large-scale analysis. In this study, GO functional annotations were obtained from the GO database [42]. To evaluate the statistical significance of the overlap between selected genes, we used AmiGo [43] to perform GO terms enrichment [43]. FlyBase [44] was used as a database filter in the GO analysis.

### Boolean network modeling and simulation analysis

In a Boolean model, at any given time (*t*), each node (*i*) has only two states: $S_{i}(t)=0$ and $S_{i}(t)=1$. For given states $S_{i}(t)(i=1\text{,}\;2\text{,}....\text{,}\;n)$ at time *t*, the next state $S_{i}(t+1)$ is determined by the following simple rule [45]:

(1) $$\left\{ \begin{array}{c} 1\text{,}\;\sum_{j=1}^{n}a_{\mathrm{ij}}S_{j}(t)>0\\ 0\text{,}\;\sum_{j=1}^{n}a_{\mathrm{ij}}S_{j}(t)<0\\ S_{i}(t)\text{,}\;\sum_{j=1}^{n}a_{\mathrm{ij}}S_{j}(t)=0 \end{array}\right.$$

where $a_{\mathrm{ij}}$ is given as follows:

(2) $$a_{\mathrm{ij}}=\{\begin{array}{l} \;0,\text{ if node }j\text{ does not regulate node }i\\ \;1,\text{ if node }j\text{ activates node }i\\ -1,\text{ if node }j\text{ represses or inhibits node }i \end{array}$$

## Competing interests

The authors have declared that no competing interests exist.

## Authors’ contributions

KHC designed the study, MSK performed the simulations, MSK, JRK, DK, ADL and KHC analyzed the data, and MSK and KHC wrote the paper. All authors read and approved the final manuscript.

## Supplementary Material

Additional file 1: **This file includes Additional Figure S1, Figure S2 [**[40]**], Figure S3 [**[6]**], and Supplementary Tables S1.** Click here for file

Additional file 2: **This file includes the interactions in the gene regulatory network of *Drosophila melanogaster.*** Click here for file
