# Supplementary material for: Spatiotemporal network motif reveals the biological traits of developmental gene regulatory networks in Drosophila melanogaster
Source: BMC Syst Biol. 2012 May 1;6:31. doi: 10.1186/1752-0509-6-31 (PMC3434043; doi:10.1186/1752-0509-6-31)
Supplement: Additional file 1: — This file includes Additional Figure S1, Figure S2 [[40]], Figure S3 [[6]], and Supplementary Tables S1. [file 1752-0509-6-31-S1.doc]

**Spatiotemporal network motif reveals the biological traits of developmental gene networks in *Drosophila melanogaster***

Man-Sun Kim^1^, Jeong-Rae Kim^1,2^, Dongsan Kim^1^, Arthur D. Lander^3^,
and Kwang-Hyun Cho^1^*

^1^Department of Bio and Brain Engineering, Korea Advanced Institute of Science and Technology (KAIST), Daejeon 305-701, Republic of Korea.

^2^Department of Mathematics, University of Seoul, Seoul 130-743, Republic of Korea.

^3^Department of Developmental and Cell Biology and Department of Biomedical Engineering, Center for Complex Biological Systems, University of California, Irvine, Irvine, CA 92697-2300, USA

**Supplementary Material**


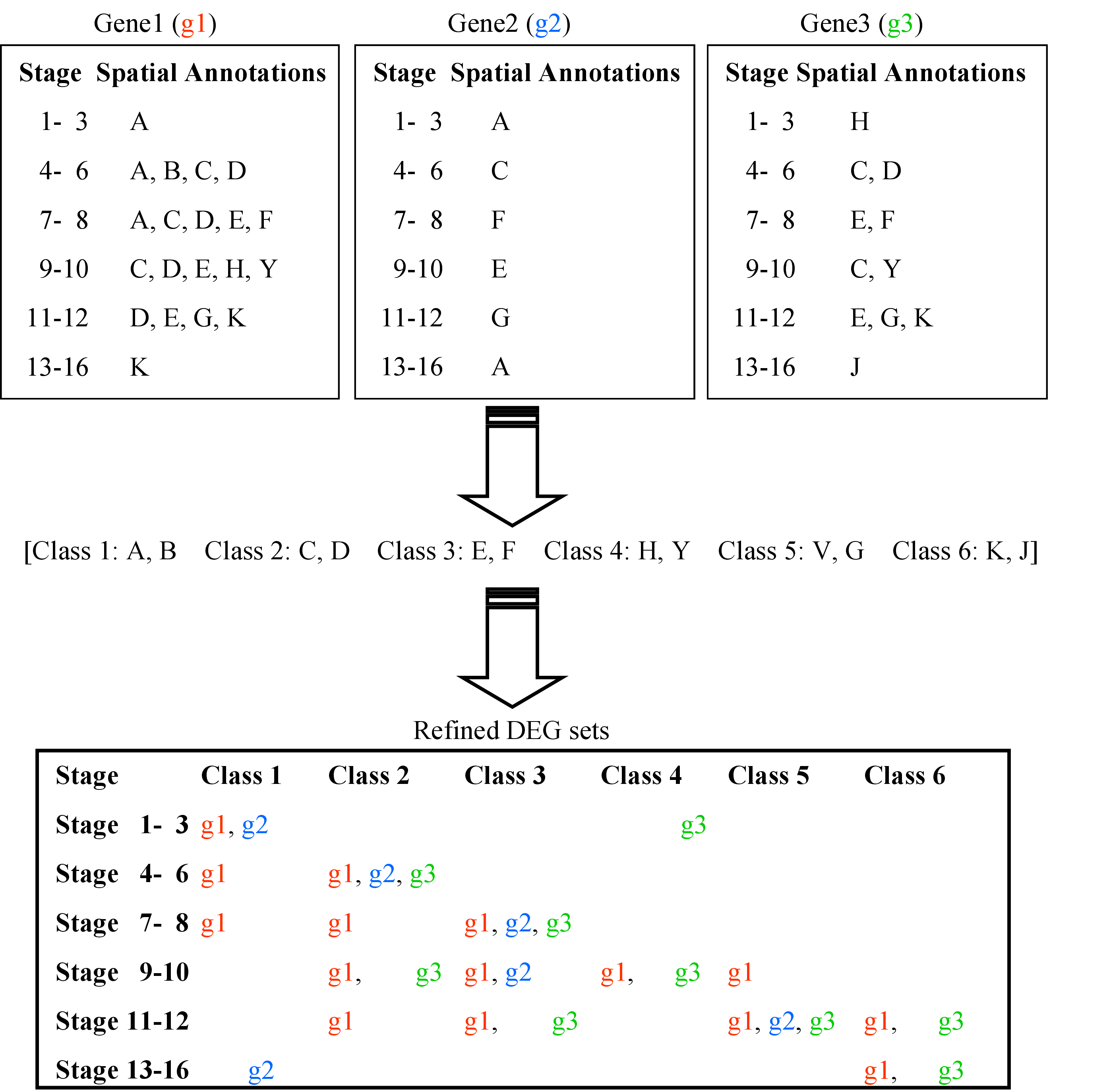


**Figure S1**. Illustration of the procedure for obtaining a refined DEG set. Each gene can be expressed at different developmental stages or spatial annotations (upper box). Since there are too many spatial annotations to deal with at the same time, we used the spatial annotations of Tomancak et al. (Tomancak et al. 2007). According to the procedure, each gene expression pattern can be refined (lower box).


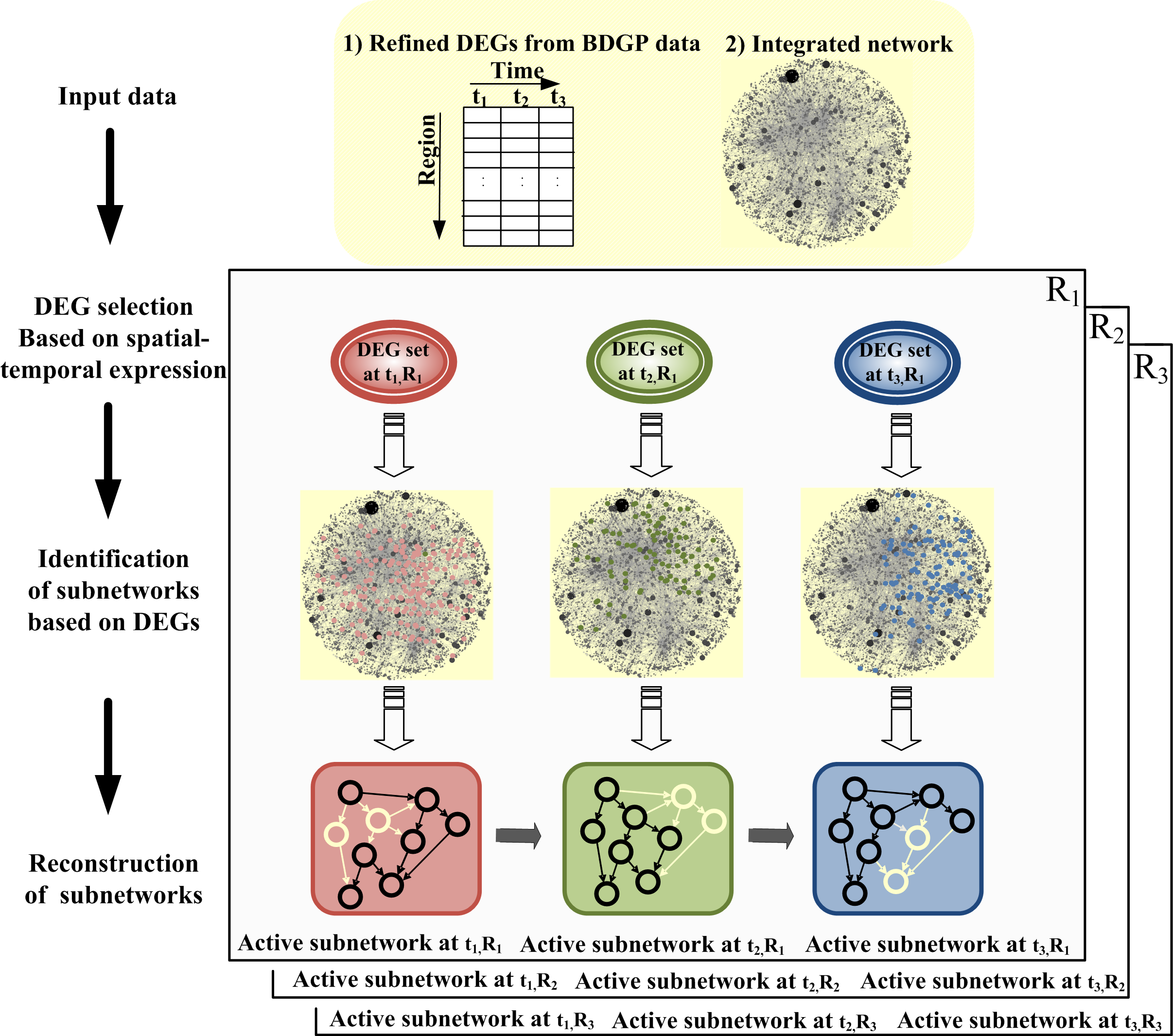


**Figure S2**. Reconstruction of active subnetworks in three regions (R_1_, R_2_ and R_3_) and three time points (t_1_, t_2_ and t_3_). In the reconstructed sub-networks, black arrows and circles denote active links and nodes, respectively; similarly white arrows and circles denote inactive links and nodes (Kim et al. 2010).


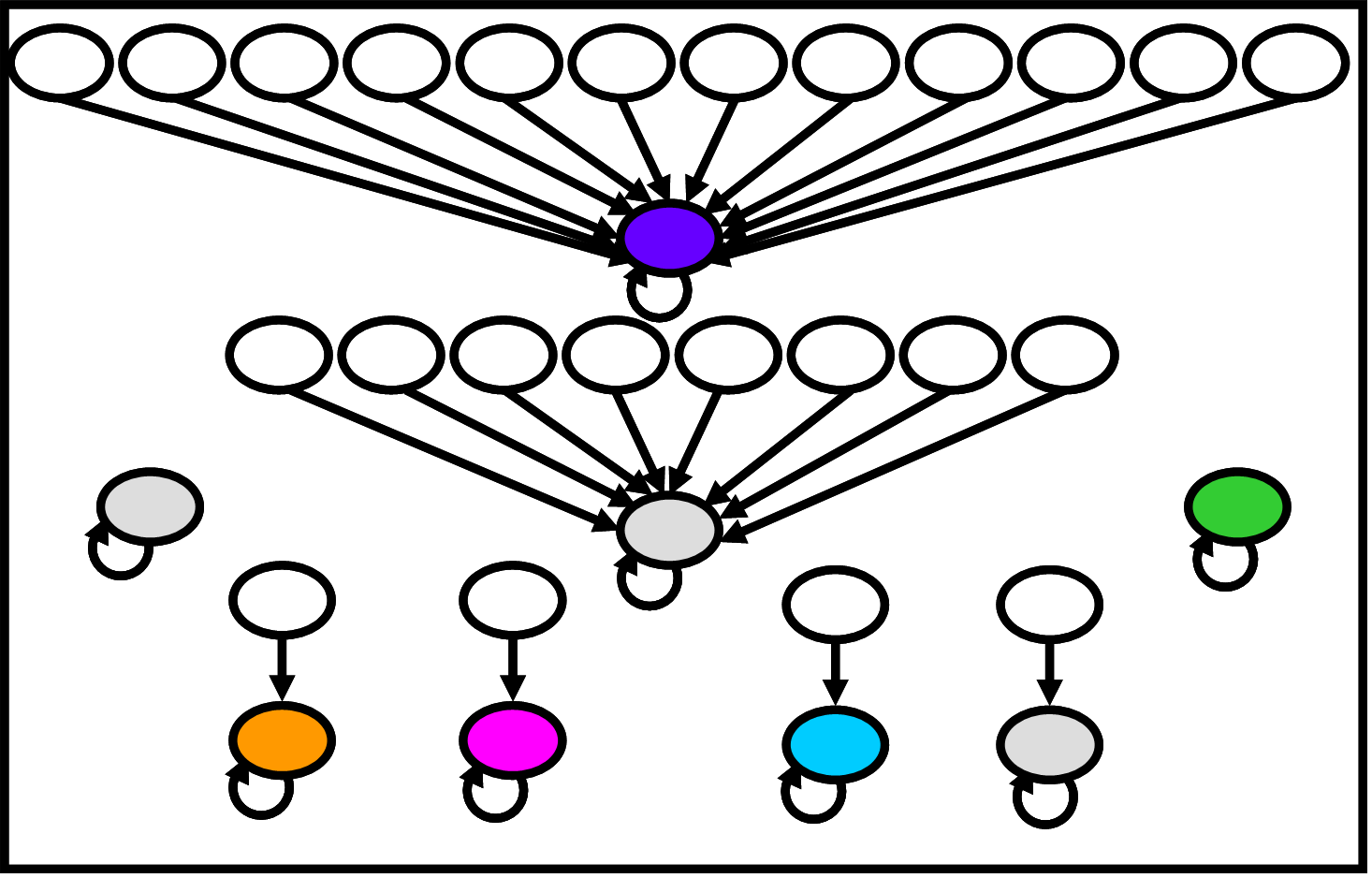


**Figure S3**. State transition map of the gap gene network. The filled circles denote attractors. The five colors in Fig. 3C correspond to five states.

**Table S1. State transition table of the gap gene network**

| **State _previous_🡪State _next_** |
| --- |
| 10010-->00000  11010-->00000  00000-->00000  01100-->00000  10100-->00000  11100-->00000  01110-->00000  10110-->00000  11110-->00000  11000-->10000  10000-->10000  01000-->01000  01010-->01000  00100-->00100  00010-->00010  00110-->00010  01001-->00001  00001-->00001  10011-->00001  11011-->00001  00011-->00001  01011-->00001  10101-->00001  11101-->00001  01101-->00001  00111-->00001  01111-->00001  11111-->00001  10111-->00001  10001-->10001  11001-->10001  00101-->00101 |

**Supplementary References**

Kim, M.S., Kim, J.R., and Cho, K.H. 2010. Dynamic network rewiring determines temporal regulatory functions in Drosophila melanogaster development processes. *Bioessays* **32**(6): 505-513.

Tomancak, P., Berman, B.P., Beaton, A., Weiszmann, R., Kwan, E., Hartenstein, V., Celniker, S.E., and Rubin, G.M. 2007. Global analysis of patterns of gene expression during Drosophila embryogenesis. *Genome Biol* **8**(7): R145.
